# Building Pathways Into Governmental Public Health Careers Through Academic-Governmental Public Health Partnerships

**DOI:** 10.3389/phrs.2024.1606780

**Published:** 2024-01-22

**Authors:** Kinsey Mannebach, Ruby H. N. Nguyen, Deborah Radi, Isabel J. Ricke, Mickey Scullard, Elizabeth V. Wattenberg

**Affiliations:** ^1^ School of Public Health, University of Minnesota, Minneapolis, MN, United States; ^2^ Minnesota Department of Health, Saint Paul, MN, United States

**Keywords:** public health workforce, emergency preparedness, experiential learning, academic-governmental public health partnership, internships

## Introduction

Governmental public health in the state of Minnesota (in the United States), as in many other U.S. states, needs to increase its workforce, given its depletion leading up to, and following, the COVID-19 pandemic [1]. To address this, the Minnesota Department of Health (MDH) and the University of Minnesota School of Public Health (UMN SPH) formed a partnership to develop a program to broaden the pathways to careers in governmental public health for undergraduate, graduate, and Master of Public Health (MPH) students.

Minnesota, located in the north central U.S., population approximately 5.7 million, is roughly the size of the United Kingdom. In the U.S., states are primarily responsible for protecting the public health of their residents. The Minnesota public health structure consists of a partnership between MDH, 11 sovereign tribal nation health departments, which serve American Indian communities, and over 50 community health boards. MDH and UMN SPH are located in the major urban center of Minnesota, while many of the local and tribal public health agencies are located in rural regions. Among the challenges that Minnesota public health agencies face are recruiting and retaining staff, and maintaining readiness for response to emergencies, including those caused by human action, natural disasters, and infectious diseases.

**FIGURE 1:**
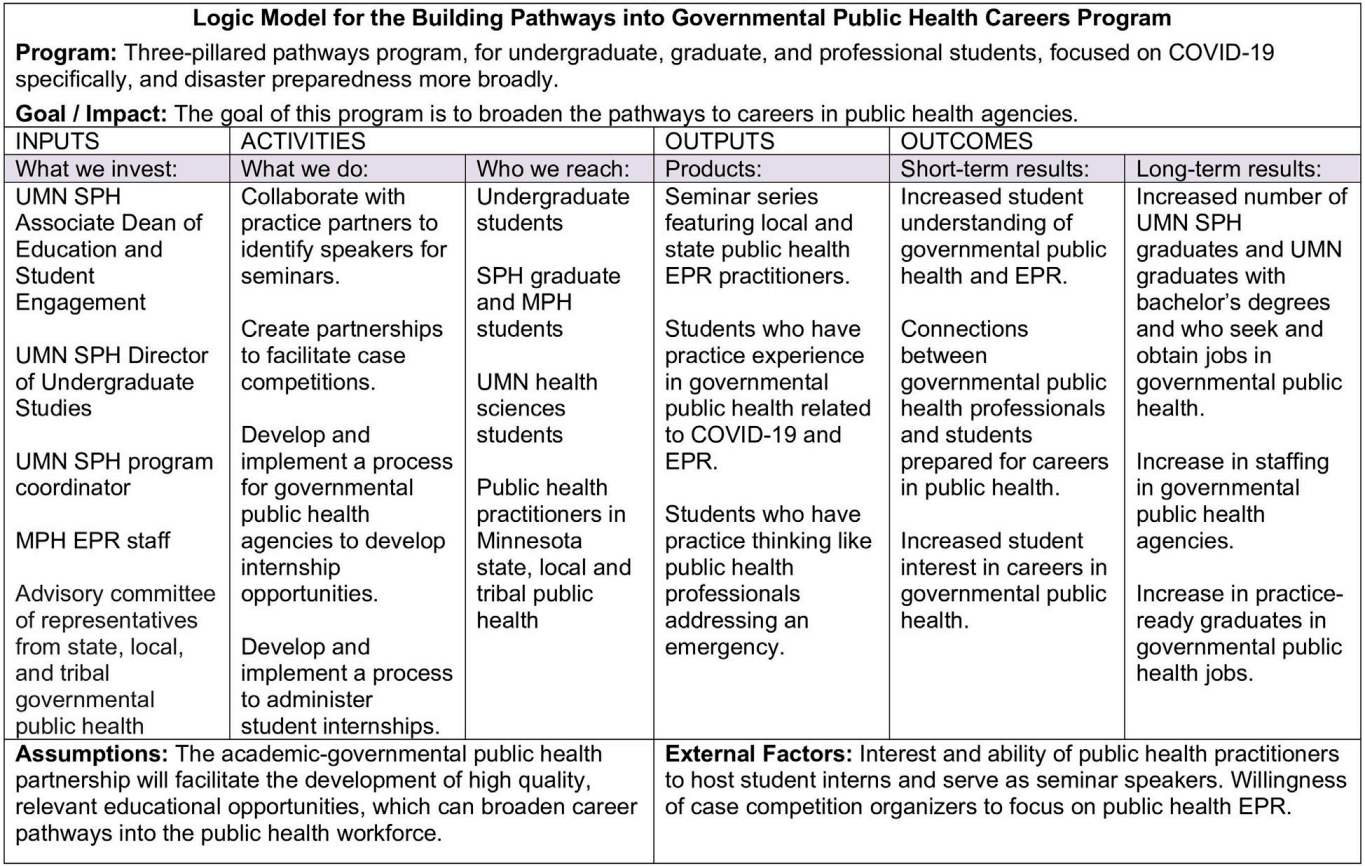
Logic model for the building pathways into governmental public health careers program. (Public Health Crisis Response Cooperative Agreement: Academic Partnership to Enhance Workforce Preparedness, United States, 2022–2024). University of Minnesota School of Public Health (UMN SPH); Minnesota Department of Health (MDH); Emergency Preparedness and Response (EPR).

MDH received a federal grant from the Centers for Disease Control and Prevention, which focuses on COVID-19 crisis workforce development and emergency preparedness and response (EPR). This grant funded and guided the development of our three-pillared program (Figure 1). MDH and UMN SPH program partners share governance of the program; decisions are made through discussion and group consensus. The program partners also consult regularly with an advisory group of local, tribal, and state public health professionals. This academic-governmental public health partnership offers an approach to rebuild the essential governmental public health workforce.

## Pillar One: Seminar Series

Starting with undergraduates, we wanted to inspire students and encourage them to consider careers in governmental public health and EPR. To reach undergraduates, we embedded a seminar series called “Stories from the Field” into an undergraduate *Introduction to Epidemiology* course. During each seminar, local and state public health EPR practitioners share their education and pathways into their current careers, how their jobs were shaped by the COVID-19 pandemic, and why they enjoy working in this field. Bringing current professionals into the classroom helps students learn about real-life scenarios and experiences, and network with potential future employers and colleagues.

Understanding that many public health professionals have numerous commitments, we work closely with our MDH colleagues and our advisory group to conduct targeted outreach and speaker recruitment. We also accommodate practitioners’ needs, including providing the option of presenting over Zoom. The seminars start with a personalized video welcome from a prominent public health practitioner with EPR experience. Each seminar features two or three guest speakers from public health agencies across Minnesota. Initially, we observed that most students lacked the foundational knowledge of governmental public health and EPR to fully absorb the speakers’ content. To address this, we now begin each semester with a seminar by an MDH expert who provides an overview of EPR.

## Pillar Two: Internship Program

We launched a paid internship program to both help with COVID-19 response and recovery work, and to provide experiential learning opportunities for undergraduate and UMN SPH graduate and MPH students. First, we consulted with our advisory group to understand the needs of the public health agencies and to identify barriers to participation. The top barriers included identifying projects, supervising students, and managing administration. To address these barriers, MDH staff worked with their colleagues to brainstorm ideas and explore creative methods for managing projects. In addition, UMN SPH conducted the administrative work of recruitment, hiring, and payroll of student interns, offered help with project development, and frequently checked in to identify and resolve any supervisor-intern issues.

Up to now, interns have worked on several projects, including COVID-19 storytelling, journal article writing, qualitative data analysis, inventory management, community engagement, and emergency plan revision. Surveys indicate that the overwhelming majority of supervisors would recommend the internship program to their public health colleagues, and would consider hosting another student intern. Likewise, surveys indicate that the majority of interns felt that their experience was meaningful; that they were highly satisfied with the support provided by their supervisor and UMN SPH; that the experiences improved their understanding of governmental public health, EPR, and COVID-19 response and recovery; and that the experience increased their interest in EPR as a potential career. There were clearly mutual benefits of the internship program: many of the internships were extended, and some students were offered positions from their internship host site.

The internship program revealed a need to develop alternative approaches for pathways into careers in local and tribal public health agencies located in rural areas. In addition to lacking the capacity to supervise students, rural agency staff were reluctant to propose a project because they were concerned that students from an urban university would not be willing or able to work on site, and because personal community connection is important. One future strategy would be to develop internship programs with colleges located closer to the rural public health agencies.

## Pillar Three: Case Competitions

Another strategy we used to build interest around governmental public health EPR careers was to offer case competitions in which interdisciplinary teams of health sciences students develop response strategies to a disaster. We partnered with university organizations who have existing case competition programs. The cases are written by a team that includes members from UMN SPH and MDH. They are developed with the following aims: Introduce a large number of undergraduate and graduate students to a community health challenge faced by Minnesotans, which governmental public health agencies need to address; Provide mentorship to students by public health professionals; Create an opportunity for students to think critically about how to solve community health needs related to COVID-19 and EPR. This activity allows students to start thinking like public health professionals by asking them to provide recommendations and propose actions to address the emergency.

Partnering with existing case competition programs increased the reach to students from a variety of academic disciplines. This improved the quality of the experience by bringing together a variety of perspectives and expertise. Surveys indicated that participants found that the competition was a valuable learning experience that increased their interest in a career in public health EPR.

## Conclusion

Grant funding helped us establish the infrastructure needed to launch and sustain this program. The seminar series and case competitions, which have minimal costs, will continue, although the focus of future cases may change. UMN SPH MPH students are required to complete an internship. Although the grant funding for internships will end, students often complete unpaid internships because they value the experience and professional connections. The processes we developed, and the positive experiences of intern supervisors, will help grow the internship opportunities in governmental public health. The short-term outcomes of this program are promising. Surveys indicate that the students gained knowledge of, and interest in careers in, governmental public health through the seminar series, internships, and case competitions. Finally, we plan to track the interns to determine if, in the long run, they apply for and obtain employment in governmental public health.
